# Oligodendrocyte precursor cells engulf synapses during circuit remodeling in mice

**DOI:** 10.1038/s41593-022-01170-x

**Published:** 2022-09-28

**Authors:** Yohan S. S. Auguste, Austin Ferro, Jessica A. Kahng, Andre M. Xavier, Jessica R. Dixon, Uma Vrudhula, Anne-Sarah Nichitiu, Daniele Rosado, Tse-Luen Wee, Ullas V. Pedmale, Lucas Cheadle

**Affiliations:** 1 Cold Spring Harbor Laboratory, Cold Spring Harbor, NY USA; 2 School of Biological Sciences, Cold Spring Harbor Laboratory, Cold Spring Harbor, NY USA

**Keywords:** Glial biology, Synaptic development, Glial stem cells

## Abstract

Oligodendrocyte precursor cells (OPCs) give rise to myelinating oligodendrocytes throughout life, but the functions of OPCs are not limited to oligodendrogenesis. Here we show that OPCs contribute to thalamocortical presynapse elimination in the developing and adult mouse visual cortex. OPC-mediated synapse engulfment increases in response to sensory experience during neural circuit refinement. Our data suggest that OPCs may regulate synaptic connectivity in the brain independently of oligodendrogenesis.

## Abstract (web summary)

Oligodendrocyte precursor cells (OPCs) have functions beyond oligodendrogenesis. Here the authors show that OPCs can engulf thalamocortical presynapses in response to sensory experience in mice.

## Main

The refinement of synapses in response to sensory experience sculpts brain connectivity during late stages of postnatal development and facilitates circuit plasticity in adults^1^. However, the cellular and molecular mechanisms underlying experience-dependent refinement remain poorly understood. Recent work has demonstrated that microglia and astrocytes promote synaptic refinement in the mammalian visual system through the phagocytic engulfment of excess synapses during the first week of life^2,3^. While this discovery unveiled synapse engulfment as a core biological mechanism through which glia shape circuit connectivity before the onset of sensory experience at eye-opening (postnatal day (P)14), the possibility that glia serve as intermediaries between visual experience and synaptic refinement during late stages of development and in the adult remained to be extensively tested.

Oligodendrocyte precursor cells (OPCs) are a specialized population of glial progenitors that give rise to myelinating oligodendrocytes. Initially born in the subventricular zones of the embryonic neural tube, OPCs migrate throughout the brain and spinal cord where they continue to proliferate and differentiate into oligodendrocytes well into postnatal development^4^. Although the rate of oligodendrocyte production decreases substantially as the brain matures, OPCs remain abundant and maintain their capacity to differentiate in the adult brain. In addition to their ability to receive input from neurons at bona fide synapses^5^, the persistence of OPCs across the lifespan after mature myelination patterns have been established suggests that OPCs may have important roles in the brain beyond their contributions as a progenitor pool. Indeed, recent work suggests that OPCs can contribute to antigen presentation in vitro^6^, glial scar formation after spinal cord injury^7^, angiogenesis^8^, and axonal remodeling in zebrafish^9^. However, the functions of OPCs remain to be fully characterized.

**Fig. 1:**
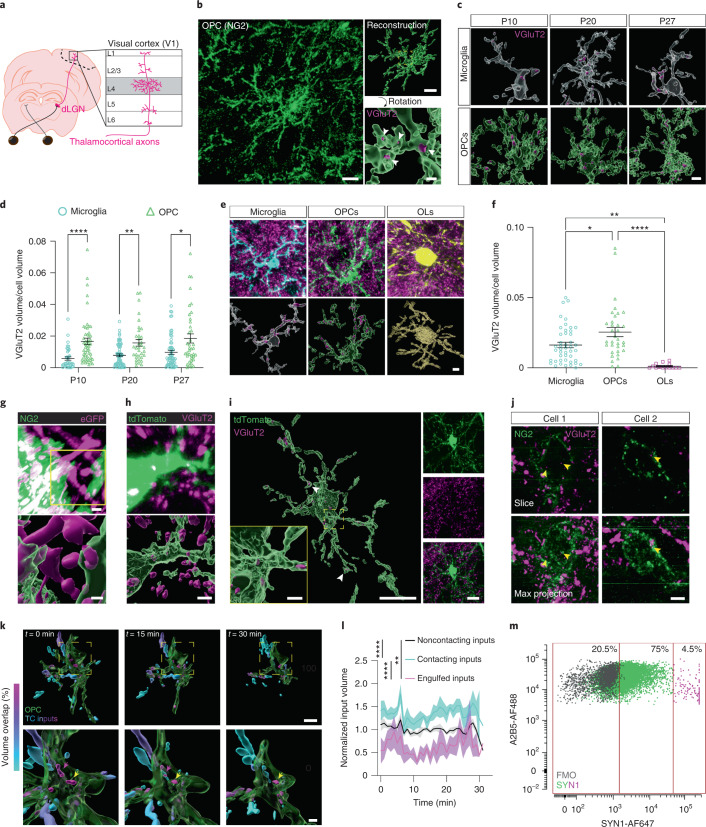
OPCs engulf TC synaptic inputs in V1. **a**, Schematic of TC inputs terminating in mouse V1. **b**, Confocal image and volumetric reconstructions of an OPC (NG2, green) containing TC inputs (VGluT2, magenta and white arrowheads). Scale bars, 10 μm, 5 μm (inset top) and 2 μm (inset bottom). **c**, Reconstructions of microglia (Iba1, white) and OPCs (NG2, green) containing TC inputs (VGluT2, magenta) during development. Scale bar, 5 μm. **d**, Quantification of synaptic material within microglia and OPCs. Two-way ANOVA with Geisser–Greenhouse correction (cell type: *P* < 0.0001; age: *P* = 0.6225; interaction: *P* = 0.1776) and Šídák multiple comparisons. *n* (microglia/OPCs): P10 = 38/53, P20 = 63/35 and P27 = 60/39, from three mice per group. **e**, Images and reconstructions of a microglia (Iba1, cyan), an OPC (green) and a mature oligodendrocyte (yellow) in an adult NG2-CreERT^2^tdTomato mouse, stained for TC inputs (VGluT2, magenta). Scale bar, 5 µm. OLs, oligodendrocytes. **f**, Quantification of the volume of synaptic material contained within microglia, OPCs and oligodendrocytes. One-way ANOVA (*P* < 0.0001) with Tukey’s posthoc test; *n* (microglia/OPCs/OLs) = 45/34/14 from three mice per group. **g**, Image and reconstruction of an OPC (NG2, pseudocolored green) containing AAV-hSYN-eGFP^+^ TC inputs (pseudocolored magenta). Scale bars, 2 μm. **h**, Image and reconstruction of an OPC (tdTomato, green) containing VGluT2-stained inputs (magenta). Scale bar, 2.5 μm. **i**, An OPC (tdTomato, green) and internalized inputs (VGluT2, magenta; white arrowheads) imaged on an Airyscan microscope. Scale bars, 10 µm, 1 µm (inset) and 10 µm (right). **j**, OPCs (NG2, green) and inputs (VGluT2, magenta; yellow arrowheads) imaged on a STED microscope. Scale bar, 2 µm. **k**, Reconstructions of an OPC (green) interacting with inputs colored based on the percentage of fluorescence overlap with the OPC (magenta 100% overlap, cyan 0% overlap). Images taken from a 30-min time-lapse session shown in Supplementary Video 1. Scale bars, 10 μm (top) and 5 μm (bottom). Yellow and magenta arrowheads indicate engulfed inputs that were present throughout or disappeared during the imaging session, respectively. **l**, Average volumes of inputs based on their contact with OPCs. Lines represent mean and shaded areas represent s.e.m. Two-tailed Friedman test (*P* < 0.0001) with Dunn’s multiple-comparison correction. *n* = 6 videos taken from three mice. **m**, Flow cytometry plot demonstrating presence of the presynaptic marker SYNAPSIN (SYN1^hi^) within OPCs (A2B5^hi^). FMO, fluorescence minus one control condition. Data points are colored based on the amount of SYN contained within each OPC. In **d** and **f**, individual data points are shown with bars representing mean ± s.e.m. *P < 0.05, **P < 0.01, ****P < 0.0001.

To uncover experience-dependent mechanisms of synaptic refinement, we analyzed interactions between glia and presynaptic thalamocortical (TC) inputs from the dorsal lateral geniculate nucleus (dLGN) of the thalamus as they synapse onto their postsynaptic targets in layer 4 of the primary visual cortex (V1) of the mouse. We chose the visual TC circuit as the basis for this study because it undergoes a well-defined period of heightened experience-dependent synaptic refinement during the third week of life and because synapse elimination in the adult contributes to vision-dependent plasticity^10,11^ (Fig. 1a). We first assessed interactions between glia and synapses across postnatal development and in the mature brain by immunostaining for proteins enriched in distinct glial types along with the TC input marker VGluT2 at P10, P20, P27 (the peak of sensory-dependent refinement) and P90, when the brain is fully mature. By quantifying these interactions using a well-established engulfment assay^2^, we identified TC inputs within microglia at all time points analyzed.

**Extended Data Fig. 1:**
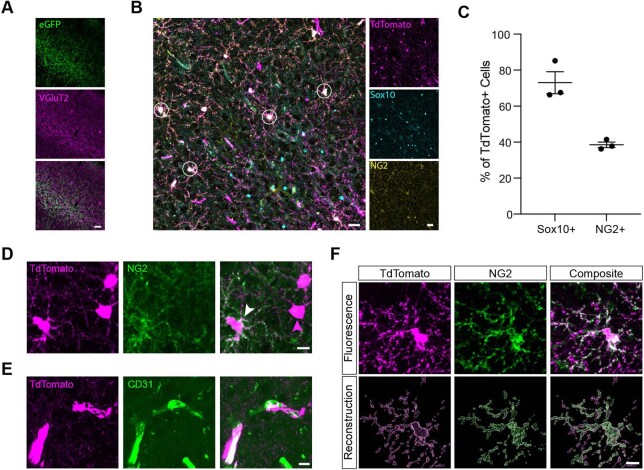
Validation of the OPC reporter line. **(a)** Confocal images of TC axons and inputs in V1 labeled with AAV-hSYN-eGFP (green) and immunostained for VGluT2 (magenta). Scale bar, 40 μm. **(b)** Representative image of tdTomato+ cells (magenta) in the NG2-CreER^T2^tdTomato mouse line. Cells of the oligodendrocyte lineage immunostained for Sox10 (cyan) and the OPC marker NG2 (yellow). White circles, OPCs confirmed by co-expression of tdTomato and NG2. Scale bar, 20 μm. Fluorescence scale bar, 40 μm. **(c)** Quantification of the percentages of tdTomato+ cells that co-stain for Sox10 and NG2. Individual data points and mean ± s.e.m. shown; *n* = 3 mice per group. **(d)** High resolution images of tdTomato+ cells (magenta) immunostained for NG2 (green). White arrow, confirmed OPC in which tdTomato and NG2 signal overlap. Compared to other tdTomato+ cells (magenta arrow), OPCs can be distinguished by their bean-shaped somata. Scale bar, 10 μm. **(e)** Confocal images of tdTomato+ cells (magenta) co-localizing with blood vessels (CD31, green). These are likely pericytes and are easy to distinguish from OPCs based upon morphology. Scale bar, 10 μm. **(f)** High magnification image of a tdTomato+ OPC (magenta) immunostained for NG2 (green). Bottom, volumetric reconstructions of the same cell based upon tdTomato versus NG2 signal, demonstrating a high level of overlap between the two channels. Scale bar, 10 μm.

**Extended Data Fig. 2:**
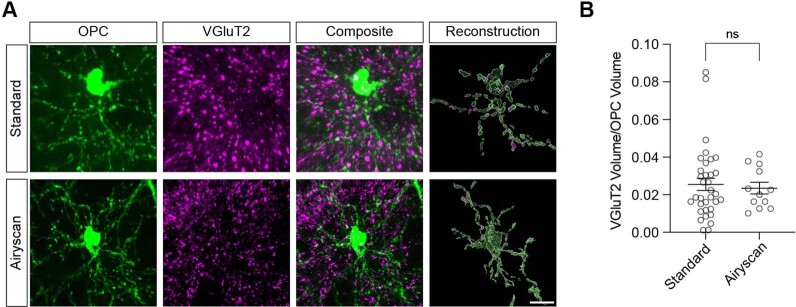
Comparison of standard and Airyscan confocal microscopy. **(a)** Fluorescence images acquired on LSM710 (top row) and LSM900 Airyscan (bottom row) confocal microscopes with a 63x oil objective. In both, OPCs from the NG2-CreER^T2^tdTomato reporter line (pseudocolored in green) were imaged along with thalamocortical inputs (VGluT2, magenta). The Airyscan example is taken from Fig. 1. Scale bar, 10 µm. **(b)** Quantification of engulfment by OPCs with both imaging techniques show similar results despite the difference in resolution. Standard confocal data is replotted from Fig. 1. Two-tailed Unpaired t-test; *P* > 0.05; *n* (standard/Airyscan) = 36/12 cells from 3 mice per group.

**Extended Data Fig. 3:**
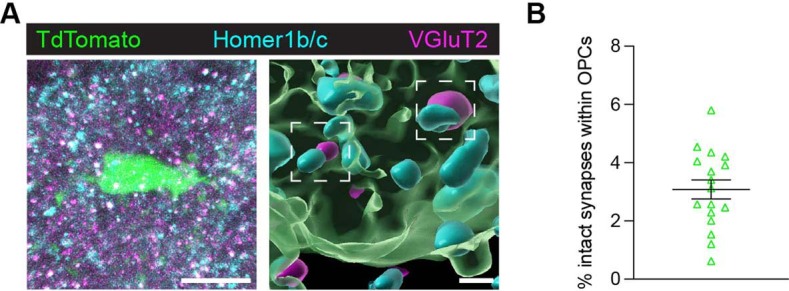
Internalization of intact synapses. **(a)** Left, confocal image of an OPC (tdTomato, green), thalamocortical (TC) inputs (VGluT2, magenta), and post-synaptic compartments (Homer1b/c, cyan). Points of colocalization between VGluT2 and Homer1b (white) represent intact synapses. Right, a reconstruction showing intact synapses within the OPC. Fluorescence scale bar, 10 µm. Reconstruction scale bar, 1 µm. **(b)** Quantification of the percentage of intact TC synapses within an OPC among all intact synapses in the imaging field. *n* = 16 cells from 3 mice. Individual data points shown with bars representing mean ± s.e.m.

**Extended Data Fig. 4:**
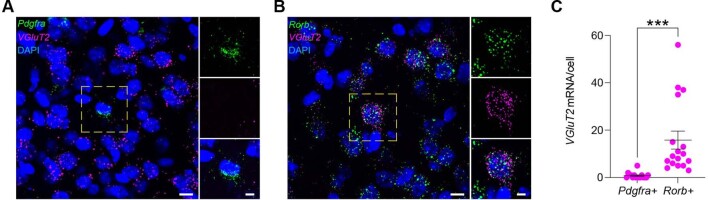
OPCs do not express *VGluT2*. Representative confocal images of V1 sections following fluorescence in situ hybridization (FISH) probed for the OPC marker *Pdgfra* **(a)** and the excitatory neuronal marker *Rorb* **(b)**, along with the mRNA encoding the presynaptic marker *VGluT2* (magenta). DAPI shown in blue. Scale bar, 10 µm. Inset scale bar, 5 µm. **(c)** Quantification of the number of *VGluT2* mRNA transcripts in each *Pdgfra* + OPC and *Rorb* + neuron in P27 mice. Unpaired two-tailed student’s T-test (*P* = 0.0005). Individual data points shown with bars representing mean ± s.e.m.; *n* (OPCs/neurons) = 16/17 from 3 mice. ***P <0.0005.

Analysis of OPCs revealed that these cells also contained TC inputs within their cellular boundaries across postnatal development and in the adult brain (Fig. 1b–d). Mature oligodendrocytes did not contain synaptic material, suggesting that the internalization of synaptic inputs is a function of OPCs that does not extend to other cells of the oligodendroglial lineage (Fig. 1e,f). Initially focusing on the mature brain, we complemented the antibody-based method for labeling presynaptic terminals ex vivo by labeling TC inputs through infection of neurons in the dLGN with AAV9-hSYN-eGFP (Extended Data Fig. 1a). Reciprocally, we labeled OPCs by crossing the NG2-CreER^T2^ mouse line^12^ with the *lox*-STOP-*lox*-tdTomato reporter line^13^ (NG2-CreER^T2^tdTomato mice; Extended Data Fig. 1b–f) and immunostained V1 sections from these mice for VGluT2. Both experiments confirmed the presence of TC inputs within OPCs (Fig. 1g,h). To gain greater resolution, we also visualized synaptic inputs within OPCs via Airyscan confocal and stimulated emission depletion (STED) microscopy (Fig. 1i,j and Extended Data Fig. 2). Finally, we observed intact synapses (that is, puncta at which VGluT2 overlapped with the postsynaptic marker Homer1b/c) within OPCs (Extended Data Fig. 3), as well as instances in which OPCs began to internalize synaptic boutons that remained in contact with axons (example in Fig. 1g). Given that OPCs do not themselves express the presynaptic marker VGluT2 (Extended Data Fig. 4), these data strongly suggest that OPCs internalize neuronal inputs in the developing and mature brain.

**Extended Data Fig. 5:**
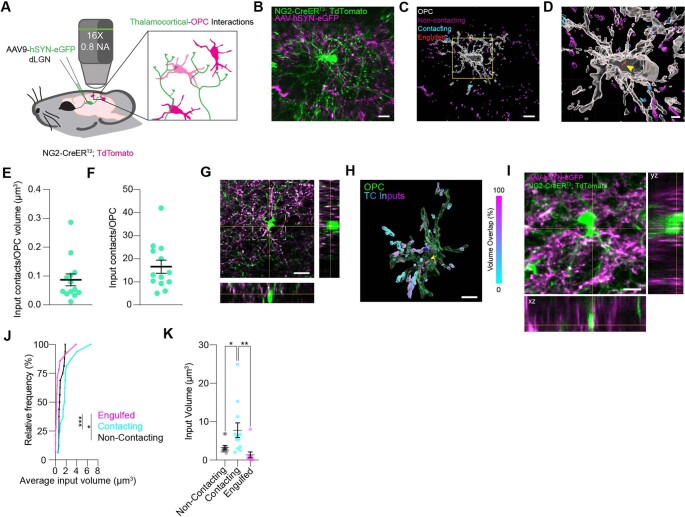
In vivo two-photon imaging of OPC-synapse interactions. **(a)** Schematic of the in vivo two-photon imaging paradigm using AAV-hSYN-eGFP to label thalamocortical (TC) inputs in the NG2-CreER^T2^tdTomato reporter line. **(b)** In vivo two-photon image of a tdTomato+ OPC (green) and eGFP+ TC inputs (magenta). Scale bar, 10 μm. **(c)** Reconstruction of OPC shown in (B) (white) with inputs color coded based upon their distance to the OPC surface (engulfed in red, contacting in cyan, and non-contacting in magenta). Scale bar, 10 μm. **(d)** Inset of reconstruction shown in (C). Yellow arrow, engulfed input. Scale bar, 3 μm. (**e,f**) The number of TC inputs contacting individual OPCs normalized to OPC volume and the number of TC inputs contacting individual OPCs (13 OPCs from 5 mice). **(g)** In vivo two-photon image of a tdTomato+ OPC and eGFP+ TC inputs including XZ and YZ orthogonal projections. Scale bar, 5 μm. **(h)** Reconstruction of OPC shown in **(g)**. OPC in green and inputs color coded based upon overlap between OPC and eGFP input signals as demonstrated by the volume overlap scale on the right. Scale bar, 3 μm. **(i)** In vivo two-photon image of a tdTomato+ OPC and eGFP+ TC inputs including XZ and YZ projections. Scale bar, 10 μm. **(j)** Cumulative frequency distribution of average input size of non-contacting, contacting, or engulfed inputs. Two-tailed Kruskal-Wallis test with Dunn’s post-hoc comparisons (engulfed vs. contacting *P* = 0.0001, engulfed vs. non-contacting *P* = 0.0354, contacting vs. non-contacting *P* > 0.05). **(k)** Quantification of the average volumes of synaptic inputs depending upon whether they contact OPCs, are engulfed by OPCs, or do not interact with OPCs. Two-tailed One-way ANOVA, *P* <0.0001  with Tukey post-hoc test (engulfed vs. contacting *P* = 0.0038, engulfed vs. non-contacting *P* = 0.5258, contacting vs. non-contacting *P* = 0.0412). *n* = 10, 12, and 12 OPCs from 5 mice for engulfed, contacting and non-contacting volumes. Data represented in histograms as mean ± s.e.m. *P < 0.05, **P < 0.01, ***P < 0.001.

To visualize synapses within OPCs in vivo, we infected the dLGNs of adult NG2-CreER^T2^tdTomato mice with AAV9-hSYN-eGFP and then imaged interactions between OPCs and TC inputs in layers 3 and 4 of V1 in awake, head-fixed mice by two-photon microscopy (Extended Data Fig. 5a). First, we captured single-time-point volumes of V1 containing tdTomato^+^ OPCs interacting with eGFP^+^ TC inputs and quantified the number of synaptic inputs that interacted with each OPC in the field of view. Consistent with previous reports that OPCs receive synaptic input from neurons^5^, we found that every OPC was in direct contact with at least one eGFP^+^ TC input and that each OPC interacted with 16.5 ± 2.85 inputs on average (Extended Data Fig. 5b–f). Furthermore, we observed that a large majority (88.5%) of OPCs contained eGFP^+^ material. Time-lapse imaging of interactions between TC inputs and OPCs over a 30-min period revealed that inputs engulfed by OPCs were smaller than inputs contacting the OPC surface and that internalized inputs remained small and relatively stable across this time frame (Fig. 1k,l, Extended Data Fig. 5g–k and Supplementary Videos 1 and 2). These data indicate that OPCs internalize TC inputs in vivo.

**Extended Data Fig. 6:**
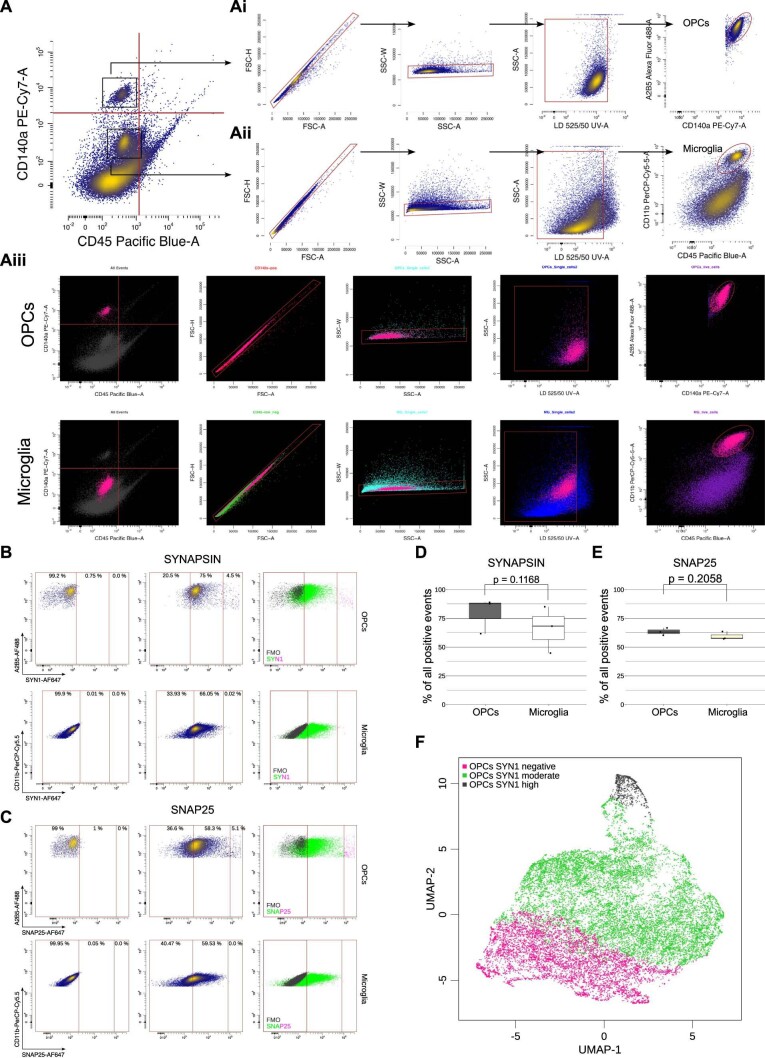
Analysis of synaptic engulfment by OPCs and microglia using flow cytometry. **(a)** Representative gating strategy for OPCs and microglia. The cells were initially distinguished from debris by the expression of CD45 and PDGFRA proteins. Next, the CD45low and PDGFRAhi population were separately gated as follows: (Ai) single cells1 →single cells2 →viable cells →OPCs (PDGFRAhi+A2B5hi) and (Aii) microglia (Cd11bhi+CD45low). (Aiii) Back gating strategy was used as a reference for OPCs and microglia population between the different samples. *n* = 3 mice at P55. **(b)**,(**c**) Flow cytometry plots showing immunoreactivity for SYNAPSIN and SNAP25 in both OPCs and microglia. An independent fluorescence minus one (FMO) control (grey) was used as a baseline to set the gate for the negative (SYN1/SNAP25 – negative) and positive (SYN1/SNAP25 – moderate and SYN1/SNAP25 – high) events. The highest mean fluorescence intensity in the microglia population was used to set the gate for the ‘high engulfers’ in OPCs (SYN1/SNAP25 – high, highlighted in magenta on the merged plot). **(d)**,(**e**) Relative quantification of all positive events for SYN1 and SNAP25 in OPC and microglia populations. Two-tailed paired t-test, *P* > 0.05. Box plots show s.e.m., 25% quartile and median values. (**f**) UMAP showing a distinct OPC population with high immunoreactivity for SYN1 compared to the negative and moderate populations. All data represented is pooled data from three independent biological replicates.

While microscopy is a common method through which synaptic engulfment is assessed, this approach allows for the sampling of only a relatively small number of cells. To investigate the possibility that OPCs engulf synapses in a heterogeneous fashion, we established a higher-throughput method for quantifying the amount of presynaptic material (that is, the proteins SYNAPSIN1 and SNAP25) in a much larger number of OPCs using flow cytometry^14^. This approach revealed that, among 25,094 cortical OPCs profiled, about 20% of OPCs did not contain appreciable amounts of synaptic material, about 75% of OPCs contained a moderate amount of synaptic material and about 5% of OPCs contained a large amount of synaptic material (Fig. 1m and Extended Data Fig. 6). These data suggest that OPCs are heterogeneous in the degree to which they engulf and/or degrade synaptic inputs, and/or that our experiment captured OPCs at multiple stages of engulfment.

**Fig. 2:**
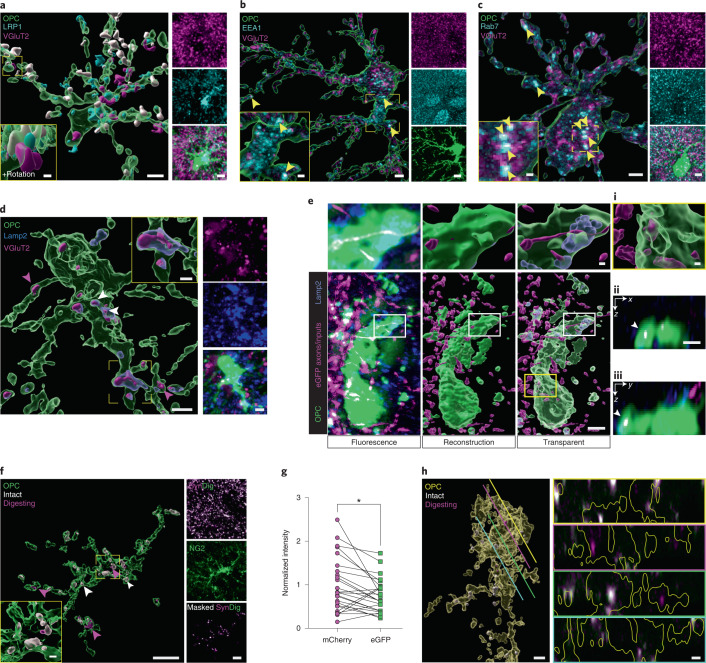
Inputs engulfed by OPCs are degraded within phagosomal compartments. **a**, Confocal images and reconstructions of an OPC (tdTomato, pseudocolored green), LRP1 (cyan) and TC inputs (VGluT2). VGluT2 inputs with LRP1 in magenta, without LRP1 in white. Scale bars, 5 µm, 1 µm (inset) and 5 µm (fluorescence). **b**, Images and reconstruction of an OPC (tdTomato, green) containing the early phagosomal marker EEA1 (cyan) and TC inputs (VGluT2, magenta). Yellow arrowheads, colocalization between VGluT2 and EEA1. Scale bars, 2 µm, 1 µm (inset) and 4 µm (fluorescence). **c**, Reconstruction of an OPC (tdTomato, green) containing the late phagosomal marker Rab7 (cyan) and TC inputs (VGluT2, magenta). Yellow arrowheads, colocalization between VGluT2 and Rab7. Scale bars, 2 µm, 0.5 µm (inset) and 2 µm (fluorescence). **d**, Images and reconstruction of an OPC (tdTomato, green) containing TC inputs (VGluT2, magenta) and lysosomes (Lamp2, blue). White arrowheads, colocalization between VGluT2 and Lamp2. Scale bars, 2 µm, 1 µm (inset) and 2 µm (fluorescence). **e**, Images of an OPC (tdTomato, green), TC inputs (AAV-hSYN-eGFP, magenta) and Lamp2 (blue) taken on a structured illumination microscope alongside reconstructions. Scale bars, 16 μm, 1 μm (inset). (i), Increased magnification of inputs outside of Lamp2. Scale bar, 1 μm. (ii,iii), Orthogonal views of the OPC (green) containing inputs (white). Scale bar, 1 μm. **f**, Images and reconstructions of an OPC (NG2, green) containing TC inputs expressing AAV-hSYN-pSynDig (magenta and white) and an image of the pSynDig-expressing inputs within the volume of the OPC. Scale bars, 10 μm and 1 µm (inset). **g**, Quantification of pSynDig eGFP and mCherry signals within OPCs. Two-tailed ratio paired *t*-test, *P* = 0.0485; *n* = 25 cells from three mice. **h**, Images and reconstruction of an OPC (yellow) and pSynDig fluorescence signal (intact inputs, white; inputs being digested, magenta) taken on an Airyscan microscope. Lines demonstrate the location along the reconstructed OPC from which the cross-section image on the right was taken. In panels on the right, the OPC volume is outlined in yellow. Scale bars, 2 μm and 1 μm (cross-sections). *P < 0.05.

**Extended Data Fig. 7:**
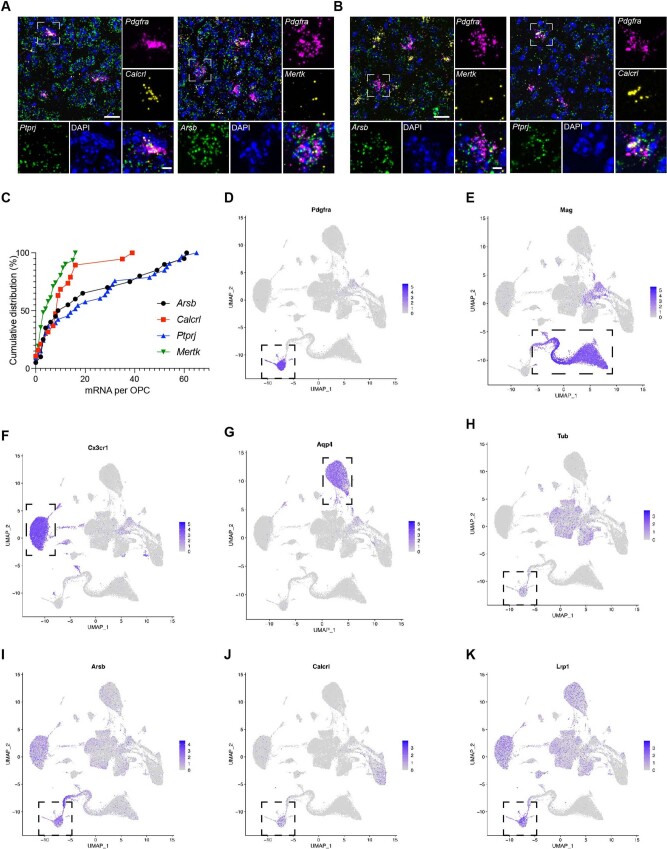
Expression of engulfment- and phagocytosis-related genes by OPCs. **(a)**,**(b)** Confocal images of V1 sections following fluorescence in situ hybridization (RNAscope) probed for the OPC marker gene *Pdgfra* (magenta) along with genes that encode known regulators of phagocytic engulfment and lysosomal function: *Ptprj* (green), *Calcrl* (yellow), *Arsb* (green), and *Mertk* (yellow). DAPI shown in blue. Sections from mice at P27 (**a**) or P90 (**b**). Scale bar, 20 μm. Inset scale bar, 5 μm. (**c**) Quantification of the number of mRNA molecules representing the genes visualized in (A) and (B) per *Pdgfra* + OPC at the P27 time point. Data plotted as the cumulative frequency distribution representing the percentage of OPCs (y-axis) expressing a given number of mRNAs by gene (x-axis). *n* = 20 OPCs (*Arsb*), 19 OPCs (*Calcrl*), 33 OPCs (*Ptprj*), and 31 OPCs (*Mertk*) from three mice per condition. (**d**)–(**g**) Uniform Manifold Approximation and Projection (UMAP) plots demonstrating cluster markers for OPCs (*Pdgfra*, D), mature oligodendrocytes (*Mag*, E), microglia (*Cx3cr1*, F), and astrocytes (*Aqp4*, G) in the single-cell dataset from Hrvatin et al., 2018. (**h**)–(**k**) UMAPs demonstrating the expression of engulfment-related genes across cell types. Note the shared expression of the genes between OPCs and other cell types that engulf synapses, including microglia and astrocytes. Also note the enrichment of each gene in OPCs compared to mature oligodendrocytes, which do not engulf synapses (see Fig. 1E,F).

**Extended Data Fig. 8:**
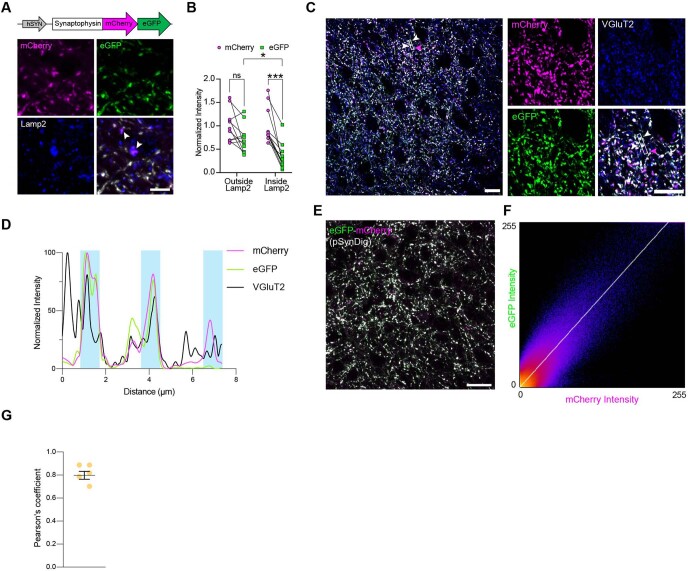
Validation of pSynDig as a marker of synaptic inputs. (**a**) Schematic of the AAV-hSYN-pSynDig virus and confocal images demonstrating the quenching of eGFP fluorescence selectively within Lamp2+ lysosomes (white arrows). Scale bar, 5 μm. (**b**) Quantification of the mCherry and eGFP signal at inputs outside of and within lysosomes normalized to the mCherry signal. The eGFP signal was significantly decreased compared to mCherry signal only at inputs within lysosomes. Connected points represent data from one image. Two-tailed, two-way ANOVA (Signal: *P* > 0.05; Interaction between signal and localization: *P* > 0.05) with Tukey post-hoc test. *n* = 11 images from 3 mice. (**c**) Confocal image of layer 4 of V1 following viral infection of the dorsal lateral geniculate nucleus (dLGN) with AAV9-hSYN-pSynDig. Most pSynDig+ inputs show tight colocalization with the input marker VGluT2 (blue). White box, example region quantified in (**d**). White arrows, inputs that are positive for both mCherry and eGFP. Magenta arrow, input that is only positive for mCherry. Scale bar, 10 μm. Inset scale bar, 10 μm. (**d**) Quantification of fluorescence intensity across the line scan denoted by the white box in (**c**) for each channel separately, normalized to each channel’s respective maximum intensity. Note the high degree of overlap between mCherry and eGFP signal in the first two blue bars and mCherry alone in the last bar. Also note the presence of a VGluT2+ synapse represented by the black peak that precedes the first blue bar, which is likely derived from a dLGN neuron that was not infected with the pSynDig virus. (**e**) Confocal image of pSynDig+ inputs in V1. Scale bar, 20 μm. (**f**) Quantification of fluorescence intensities of eGFP and mCherry across the imaging frame shows a high degree of colocalization. (**g**) Quantification of Pearson’s coefficient describing the colocalization of mCherry and eGFP signal at inputs expressing pSynDig. Individual data points shown with bars representing mean ± s.e.m. *n* = 5 images from 3 mice.

We next asked whether, like microglia, OPCs internalize synapses through phagocytosis. We found that a large number of OPCs express the molecular machinery necessary to phagocytose extracellular material, including the phagocytic receptor LRP1, which we observed at about 30% of synapses contacted by OPCs (Fig. 2a and Extended Data Fig. 7). Consistent with OPCs phagocytosing synapses, VGluT2^+^ inputs within OPCs colocalized with markers of early phagosomes (EEA1), late phagosomes (Rab7) and phagolysosomes (Lamp2; Fig. 2b–e). Furthermore, we used a viral probe for synaptic digestion, AAV9-hSYN-pSynDig^15^, to demonstrate that synapses engulfed by OPCs are likely degraded within acidic lysosomal compartments (Fig. 2f–h and Extended Data Fig. 8). Altogether, these data suggest that OPCs engulf and digest synapses at least in part through phagocytic mechanisms.

**Fig. 3:**
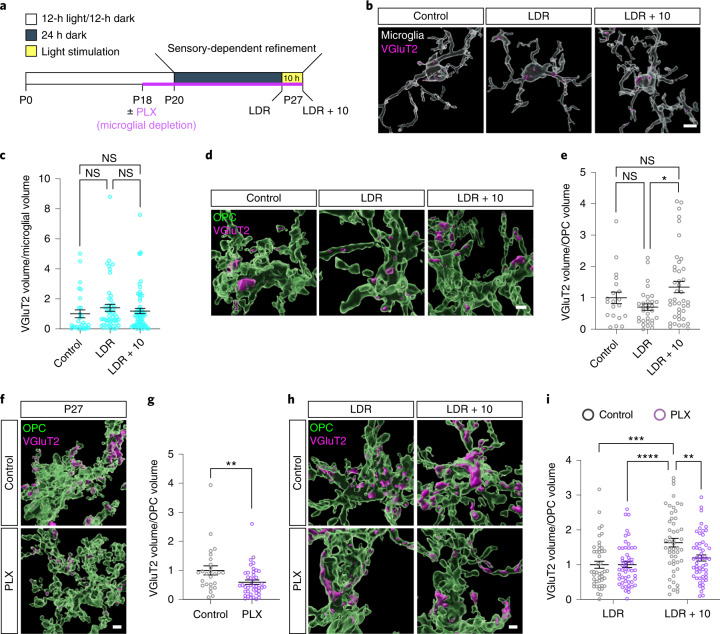
The engulfment of synaptic inputs by OPCs is heightened by sensory experience and dampened by microglial depletion. **a**, Schematic of the LDR visual deprivation and stimulation paradigm. **b**, Volumetric reconstructions of microglia (Iba1, white) and engulfed VGluT2^+^ inputs (magenta) in normally reared mice at P27 (control), mice dark-reared between P20 and P27 (LDR) and mice re-exposed to light for 10 h following LDR (LDR + 10). Scale bar, 2 μm. **c**, Quantification of the volume of synaptic material within microglia from each condition. One-way ANOVA (*P* > 0.05; NS, not significant); *n* (P27/LDR/LDR + 10): 28/49/64, from three mice per group. **d**, Reconstructions of OPCs (NG2, green) and engulfed synaptic inputs (VGluT2, magenta) from control, LDR, and LDR + 10 mice. Scale bar, 2 µm. **e,** Quantification of the volume of synaptic material contained within each OPC from control, LDR, and LDR + 10 mice. One-way ANOVA (*P* = 0.0199) with Tukey’s posthoc test; *n* (P27/LDR/LDR + 10): 20/30/42, from three mice per group. **f**, Reconstructions of OPCs (NG2, green) and engulfed TC inputs (VGluT2, magenta) in P27 mice following depletion of microglia using PLX5622 for 1 week. Scale bar, 2 μm. **g**, Quantification of the volume of synaptic material contained within each OPC in the presence or absence of microglia. Two-tailed Mann–Whitney *t*-test, *P* = 0.0082; *n* (control/PLX): 26/42, from three mice per group. **h**, Reconstructions of OPCs (NG2, green) and synaptic inputs (VGluT2, magenta) from LDR and LDR + 10 mice fed PLX5622 or control chow between P20 and P27. Scale bar, 2 μm. **i**, Quantification of synaptic engulfment in LDR and LDR + 10 mice containing or lacking microglia. Two-way ANOVA (stimulation: *P* < 0.0001; treatment: *P* < 0.05; interaction between stimulation and treatment: *P* < 0.05); *n* (OPCs, control/PLX): LDR = 45/52, LDR + 10 = 51/54, from three mice per group. In **c**,**e**,**g**, and **i**, individual data points are shown with bars representing mean ± s.e.m. *P < 0.05, **P < 0.01, ***P < 0.001 and ****P < 0.0001.

**Extended Data Fig. 9:**
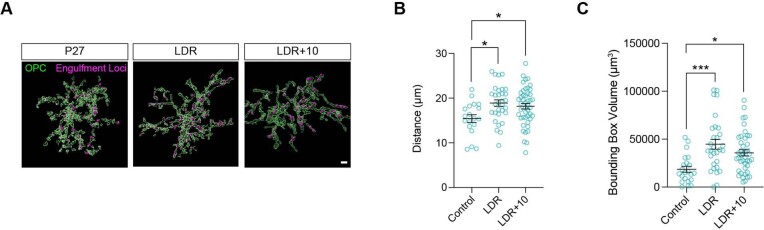
Distribution of engulfment loci across OPCs is altered in response to sensory experience. (**a**) Reconstructions of OPCs (NG2, green) and engulfment loci (VGluT2, magenta) illustrating experience-dependent changes in the distribution of points of engulfment across the OPC arbor. Scale bar, 3 μm. (**b**) Quantification of the distance between the center of the OPC soma and loci at which engulfed inputs reside. Two-tailed one-way ANOVA (*P* = 0.0131) with Tukey’s post-hoc test; n (OPC; P27/LDR/LDR + 10): 19/31/46, from 3 mice per group. (**c**) Estimated range of synaptic surveillance by a given OPC based upon the bounding box volume calculated from the distribution of engulfment loci as quantified in (b). Two-tailed, one-way ANOVA (*P* = 0.0003) with Tukey’s post-hoc test; (OPC; P27/LDR/LDR + 10): 22/30/42, from 3 mice per group. *P < 0.05. ***P < 0.001.

The observation that OPCs engulf synapses during a critical period of sensory-dependent refinement (P20–P27) suggested that they might contribute to the elimination of synapses in response to experience, which is known to occur during this period^1^. To assess this possibility, we reared mice in complete darkness between P20 and P27 (late-dark-rearing (LDR)) and then acutely re-exposed them to light for 10 h (LDR + 10; Fig. 3a). This is a widely used paradigm that activates robust patterns of sensory-driven neural activity in V1 (refs. ^16,17^). We found that microglia do not change their level of engulfment as a result of sensory deprivation or stimulation (Fig. 3b,c) but that OPCs significantly heightened their levels of engulfment in response to experience (Fig. 3d,e). The distribution of engulfment sites across the OPC arbor also reorganized as a result of sensory stimulation, providing further evidence that experience may dynamically modify the engulfment activity of OPCs (Extended Data Fig. 9).

**Extended Data Fig. 10:**
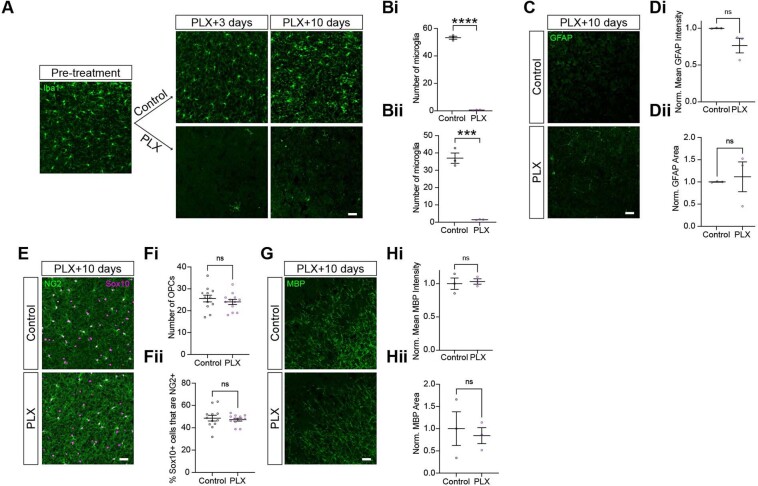
Validation of microglial depletion using PLX5622. (**a**) Representative confocal images of microglia immunostained for Iba1 (green) in the visual cortex before and during PLX5622 administration. Scale bar, 20 μm. (**b**) Quantification of the number of microglia in V1 following three days (Bi) or ten days (Bii) of PLX5622 administration. Unpaired two-tailed T test, *P* < 0.0001 . (**c**) Confocal images of V1 stained for GFAP, a marker of astrocyte activation, after ten days of PLX5622 administration. Scale bar, 20 μm. (**d**) Quantification of the mean GFAP intensity normalized to the control group (Di) and the normalized area covered by GFAP (Dii). Unpaired two-tailed T test, *P* >0.05. (**e**) Confocal images of V1 with OPCs (NG2, green) and the oligodendroglial marker Sox10 (magenta) after ten days of PLX5622 administration. Scale bar, 20 μm. (**f**) Quantification of the number of OPCs (marked by co-expression of NG2+ and Sox10+) (Fi) and the percentage of Sox10+ cells that also expressed the OPC marker NG2 (Fii). Unpaired two-tailed T test, *P* > 0.05 (**g**) Representative images of myelin (MBP, green) in mice after ten days of PLX5622 administration. Scale bar, 20 μm. (**h**) Quantification of the mean MBP intensity normalized to the control group (Hi) and quantification of the area covered by MBP (Hii). Unpaired two-tailed T test, *P* > 0.05. All graphs: Individual data points with mean ± s.e.m.; *n* = 3 mice per condition. (f) *n* = 12 images from 3 mice per condition. Individual data points shown with bars representing mean ± SEM. ***P < 0.001, ****P < 0.0001.

Given recent evidence that microglia promote the proliferation and differentiation of OPCs^18,19^, we next asked whether signals from microglia also influence the ability of OPCs to engulf synapses. To address this question, we used an inhibitor of colony-stimulating factor receptor 1, PLX5622, to deplete microglia from the brain between P20 and P27 (Extended Data Fig. 10). While the pharmacological depletion approach has the potential to lead to additional changes in the brain beyond the removal of microglia, we reasoned that this experiment would provide an important step toward defining the roles of glial–glial interactions in OPC-mediated synaptic engulfment. We found that depletion of microglia in normally reared mice between P20 and P27 significantly decreased the amount of synaptic material found within OPCs (Fig. 3f,g). Furthermore, subjecting PLX5622-treated mice to the sensory deprivation and stimulation paradigm demonstrated that the increased engulfment of synapses by OPCs in response to experience was dampened in the absence of microglia (Fig. 3h,i). Altogether, these data demonstrate that OPCs engulf synaptic inputs in response to sensory experience during a critical period of sensory-dependent plasticity and suggest that microglia may provide signals to OPCs to promote their engulfment of synapses.

Despite a growing number of studies describing the roles of glia in synaptic refinement in the early postnatal brain, how glia remodel synaptic connectivity during later experience-dependent phases of development and in the adult remains an area of active investigation. Here we uncover a new mode through which OPCs interact with neural circuits by engulfing synaptic inputs in the visual cortex in the late postnatal mammalian brain. Our findings are consistent with the observation of axonal structures within OPCs of the mouse brain as assessed by electron microscopy and the regulation of axonal remodeling by OPCs in the zebrafish tectum^9,20^. The ability of OPCs to detect changes in sensory experience is consistent with previous reports that experience regulates oligodendrogenesis and myelination in adult mice^21^. In combination with these studies, our data suggest that experience can impact OPCs in multiple ways, not only by driving adaptive myelination but also by triggering OPCs to eliminate synapses through the engulfment of presynaptic terminals. Although it is likely that OPCs contain more synapses following sensory stimulation because they increase their engulfment activity, further work is needed to rule out the possibility that experience decreases the rate at which OPCs process engulfed synapses. Given mounting evidence that deficits in the functions of oligodendrocyte-lineage cells exacerbate neurological disorders associated with synapse loss including Alzheimer’s disease and multiple sclerosis^6,22^, our discovery that OPCs can influence synapse number through the engulfment of presynaptic inputs is likely to shed light on mechanisms of disease in the human brain.

## Methods

### Animal models

All experiments were performed in compliance with protocols approved by the Institutional Animal Care and Use Committee at Cold Spring Harbor Laboratory according to protocol 20-3. The following mouse lines were obtained from the Jackson Laboratory: C57Bl/6J (JAX, 000664), NG2-CreER^T2^ (B6.Cg-Tg(Cspg4-Cre/Esr1*)BAkik/J; JAX, 008538) and Rosa26-CAG-LsL-tdTomato (B6.Cg-Gt(ROSA)26Sor^tm14(CAG-tdTomato)Hze^/J (Ai14); JAX, 007914). NG2-CreER^T2^ mice were bred with Ai14 mice inhouse to yield NG2-CreER^T2^tdTomato mice in which OPCs are labeled with tdTomato on tamoxifen (TAM) administration. Except when noted, animals were housed in normal 12-h light:12-h dark cycles at average temperatures of 68–70 °F and 54–58% humidity. Analyses were performed on equal numbers of male and female mice at postnatal days (P)10, P20, P27 and P90. Live imaging was performed on animals between 2 and 6 months of age. No sex differences were observed in this study.

### Sensory deprivation and stimulation paradigm

C57BL/6J mice were reared according to a standard 12-h light/12-h dark cycle until P20, at which point they were weaned from their mothers and separated into experimental groups. One cohort continued to be housed in normal light conditions until P27. Two cohorts of mice were placed in a well-ventilated light-proof cabinet (Actimetrics) until P27 (LDR). One of the two cohorts subjected to LDR was collected at P27 in the dark by an investigator using night vision goggles, whereas the other was acutely re-exposed to light for 10 h and then collected. These cohorts are referred to as LDR and LDR + 10, respectively, throughout the paper.

### Plexxikon 5622 administration

Mice were fed 1,200-mg kg^−1^ irradiated freebase Plexxikon 5622-formulated chow (PLX; Research Diets, Inc.: D1110404i), which blocks colony-stimulating factor receptor 1, or control chow produced in parallel (D19101002) from P18 to P27 to pharmacologically deplete microglia from the brain. Mice were fed on the chow *ad libitum*, and the investigator provided all husbandry for the mice during treatment.

### TAM administration

TAM (Sigma, T5648) was dissolved in sunflower oil (Sigma, S5007) overnight at 37 °C with shaking to achieve a working concentration of 20 mg ml^−1^. Animals were administered a bolus of TAM at 150 mg kg^−1^ on two consecutive days at a minimum of 2 weeks before imaging and analysis.

### Immunofluorescence

Animals were anesthetized with isoflurane and perfused with ice-cold PBS followed by 4% paraformaldehyde (PFA). Brains were removed and incubated in 4% PFA overnight at 4 °C. The next day, the brains were washed by rotating in PBS three times for 10 min each and allowed to sink in 15% and then 30% sucrose at 4 °C. Brains were then embedded in optimal cutting temperature (OCT; VWR, 25608-930) and stored at −80 °C. Coronal or sagittal sections with 25-µm thickness containing V1 and dLGN were collected onto Superfrost Plus microscope slides (Thermo Fisher Scientific, 1255015) using a cryostat and stored at −80 °C. For staining, the sections were washed at room temperature for 10 min in PBS and then dried for 10 min at 60 °C. A hydrophobic barrier was drawn around the samples using an ImmEdge Pen (VWR, 101098-065). A blocking solution (5% fetal bovine serum (FBS) or normal goat serum (NGS) and 0.3% TritonX-100, in PBS) was applied for 1 h at room temperature. Next, the blocking solution was replaced with primary antibodies prepared in a probing solution (5% FBS or NGS and 0.1% Triton in PBS). Primary antibodies were incubated at 4 °C overnight in most cases and for 48 h for staining with rat anti-NG2 (1:250; Thermo Fisher Scientific, MA5-24247). Other primary antibodies used included guinea pig anti-VGluT2 at 1:1,000 (Sigma, AB2251-l); rabbit anti-Iba1 at 1:1,000 (Wako, 019-19741); rat anti-Lamp2 at 1:200 (Abcam, AB13524); rabbit anti-Sox10 at 1:100 (Abcam, AB227680); chicken anti-Homer1b/c at 1:1,000 (Synaptic Systems, 160026); chicken anti-GFAP at 1:500 (Abcam, ab4674); rat anti-MBP at 1:1,000 (Abcam, ab7349); rabbit anti-LRP1 at 1:500 (Abcam, ab92544); rabbit anti EEA1 at 1:1,000 (Abcam, ab2900); and rabbit anti-Rab7 at 1:500 (Abcam, ab137029). After primary incubation, the tissue was washed four times with washing solution (PBS adjusted to 0.1% Triton) for 10 min per wash. The following Alexafluor secondary antibodies (Invitrogen) were diluted to either 1:1,000 or 1:500 in probing solution and incubated on the tissue for 1 h at room temperature: goat anti-guinea pig 647 (A21450); goat anti-guinea pig 555 (A21435); goat anti-guinea pig 488 (A11073); donkey anti-rabbit 405 (A21450); goat anti-rabbit 488 (A11008); goat anti-rabbit 647 (A21428); goat anti-rat 405 (A48261); donkey anti-rat 488 (A21208) and goat anti-rat 647 (A21247). Following secondary incubation, the sections were washed four times with washing solution, mounted with Fluoromount-G (SouthernBiotech, 0100-01) and coverslipped.

### Confocal imaging parameters

In most cases, confocal images were acquired on an LSM 710 or LSM 780 (Zeiss) microscope with ×20/0.8 NA (air) and ×63/1.4 NA (oil) objectives. *Z*-stack images were acquired to capture OPCs and/or microglia in layer 4 of V1.

### NG2-CreER^T2^tdTomato validation

Fixed brain sections from NG2-CreER^T2^tdTomato mice were stained for Sox10 and NG2 as described above and imaged on a confocal microscope at ×20. To determine the percentage of tdTomato^+^ cells that were either within the oligodendrocyte-lineage (Sox10^+^) or putative OPCs (NG2^+^), maximum projections of the images were generated to be manually counted with the ‘Cell Counter’ plugin in ImageJ. First, markers were placed at all tdTomato^+^ cells. Next, at each tdTomato^+^ marker location, the investigator determined and tabulated whether the location was also Sox10^+^ or NG2^+^.

### PLX validation

To confirm the depletion of microglia by PLX, mice were killed 3 and 10 d after being placed on the PLX or control chow. Sections were immunostained for the microglial marker Iba1, and confocal images of the cortex were acquired with a ×20 objective. Using the ImageJ cell counter plugin, the number of cells in an image was manually counted. To ensure that the population densities of oligodendroglia and OPCs were unaffected, sections at the 10-day timepoint were stained for Sox10 and NG2. Images of ×20 with the equivalent number of *z*-stacks were taken, and the cell counter plugin was used to count the number of Sox10^+^ cells (oligodendroglia) and Sox10^+^NG2^+^ cells (OPCs). We also stained sections and analyzed mean intensities for the astrocyte marker GFAP and the mature oligodendrocyte–myelin marker MBP as described above to rule out PLX-driven changes unrelated to microglial depletion.

### Fluorescence in situ hybridization

Brains were fixed by perfusion in 4% PFA, embedded in OCT and stored at −80 °C until processing. Sections of 25-µm thickness were mounted on Superfrost Plus slides. Multiplexed single-molecule fluorescence in situ hybridization (FISH) was performed using the RNAscope platform V2 kit (Advanced Cell Diagnostics (ACD), 323100) according to the manufacturer’s protocol for fixed-frozen sections. The samples were mounted with Fluoromount-G with DAPI (SouthernBiotech, 0100-20) and coverslipped. Commercial probes obtained from ACD detected the following genes: *Pdgfra* (480661-C2), *Mertk* (441241-C3), *Calcrl* (452281-C3), *Arsb* (837631) and *Ptprj* (883051).

### FISH with fresh-frozen tissue

Brains were embedded in OCT and stored at −80 °C until processing. A Leica CM3050 cryostat was used to make sections of 20-µm thickness, which were mounted on Superfrost Plus slides. Multiplexed single-molecule FISH was performed using the RNAscope platform V1 kit (ACD, 320850) according to the manufacturer’s protocol for fresh-frozen sections. The samples were mounted with Fluoromount-G with DAPI (SouthernBiotech, 0100-20) and coverslipped. Commercial probes obtained from ACD detected the following genes: *Pdgfra* (480661-C2), *Rorb* (444271) and *VGluT2* (319171-C2 and 319171).

To quantify *VGluT2* transcripts in *Pdgfra*^*+*^ and *Rorb*^*+*^ cells, ×63 confocal images were acquired using an LSM 780 Zeiss microscope. Tissue from three mice was used and a minimum of five images were analyzed per mouse. *VGluT2* expression was quantified using an ImageJ macro built inhouse. The macro first thresholded and binarized the DAPI channel before expanding it using the dilate function. This mask was then put through a watershed filter to ensure that cells that were near each other were separated. The resulting DAPI mask was used to create regions of interest (ROIs), where each ROI was considered a single cell. The number of FISH puncta within each ROI was counted using the 3D image counter function within ImageJ. ROIs were identified according to the following criteria: ROIs containing five or more *Pdgfra* or *Rorb* molecules were considered to be positive for each of the transcripts.

### Reanalysis of single-cell RNA-sequencing data

Data from ref. ^23^ were downloaded as a raw count matrix from the Gene Expression Omnibus database (GSE102827). The data were processed via the Seurat v3 pipeline using standard parameters. OPC clusters were identified by enriched expression of *Pdgfra*. For Extended Data Fig. 7, transcripts were plotted across the Uniform Manifold Approximation and Projection (UMAP) using the FeaturePlot function in Seurat.

### Engulfment quantification

Preprocessing of ×63 images stained for OPCs and/or microglia was performed in ImageJ. First, the ‘enhance contrast’ command was run so that 0.1% of pixels would be saturated, and then, a mean filter with pixel radius of 1.5 µm was applied. Next, an ROI was drawn around a given cell, cropped into its own file and used in downstream Imaris (BitPlane) processing and analysis. In Imaris, volumetric reconstructions of the fluorescence images were created using the ‘Spots’ and/or ‘Surfaces’ objects. A surface object was used to reconstruct a cell of interest following the guided creation wizard. The investigator then deleted any discontinuous part of the surface that could not be clearly traced back to the soma of the cell, using the fluorescence as a reference. Next, this cell surface was used as the ROI to create a mask of target channels (for example, VGluT2, Lamp2), defined by the signal included within the surface. New surface objects were generated using these masks, which represent the internal contents of the cell. Across conditions within an individual biological replicate, the same creation parameters were used in generating the internalized surfaces. The volumes of the cell and internalized surfaces were collected from the statistics tab in Imaris. The internalized volume was then normalized to a cell’s volume to represent the amount of engulfment by a given cell.

In some cases, masks of the Lamp2 channel within an OPC surface were made to reconstruct lysosomes. To quantify lysosome contents, the same internalization approach was used where the lysosomal surface was treated as the ‘cell’ to mask the target channel.

To quantify the distance of engulfment loci from the center of an OPC, we first defined the center of the cell by manually placing a spots object at the soma of the reconstructed cell using the autodepth-based-on-fluorescence option. Next, a spots object of the masked VGluT2 signal was created using the wizard, with a spots-diameter of 2 µm. This approach captured regions with one or more VGluT2 puncta per spot, which are referred to as ‘loci’ in the main text. The predefined ‘shortest distance to spots’ statistic was used to measure the distances between the engulfment loci and the center of the cell. To quantify the range of synaptic surveillance by a given OPC, the axis-aligned bounding-box statistic of the engulfment locus spots was collected.

### Quantification of intact synapse contact and engulfment

NG2-CreER^T2^tdTomato tissue was stained for the presynaptic marker VGluT2 and the postsynaptic marker Homer1b/c. Images that focused on tdTomato^+^ OPCs were acquired on a confocal microscope with a ×63 objective. Next, the images were cropped to include single OPCs and transferred into Imaris. Within Imaris, the ‘Coloc’ tab was used to create a new colocalization channel—a channel depicting overlapping signals of VGluT2 and Homer1b/c. The mean intensities of the VGluT2 and Homer1b/c channels were used as thresholds when creating the colocalization channel. A spots object was then created from this new channel, representing intact (containing both pre- and postsynaptic compartments) synapses. An OPC was reconstructed using the Surface object as described above. Finally, the intact synapse spots were filtered for spots less than −0.132 µm from the OPC surface (that is, more than 1 pixel within the surface of the OPC). To estimate the percentage of all VGluT2–Homer1b/c synapses an OPC interacts with, the number of internalized synapses was divided by the total number of synapses within the cropped image.

### Structured illumination microscopy

Brains were fixed in 4% PFA and then embedded in OCT and stored at −80 °C. Sections of 25-µm thickness were subjected to immunofluorescence in a free-floating format. Sections were stained with primary and secondary antibodies as described under ‘Immunofluorescence’ above, but in larger volumes of 1 ml. Stained sections were mounted onto thickness no. 1 ½ High-performance Zeiss cover glasses (Thermo Fisher Scientific, 10474379) and then centered onto Superfrost Plus microscope slides with ProLong Gold Antifade Mountant (Thermo Fisher Scientific, P36934). Stained samples were kept at 4 °C until imaging. 3D structured illumination microscopy (3D SIM) images of fixed, stained samples were acquired using an Applied Precision V3 OMX system equipped with a ×100/1.4 NA U-PLANAPO objective (Olympus) and two Cascade II 512 EM-CCD cameras (Photometrics). Stacks of six optical sections (125-nm step) were acquired consecutively in two channels (488 nm and 593 nm) using DeltaVision software (Applied Precision). 3D super-resolution image stacks were reconstructed using SoftWorx 6.5.2 using channel-specific OTFs and Wiener filter settings of 0.001 or 0.002. These image stacks were then imported into Imaris for volumetric reconstructions.

### Airyscan imaging

Samples were imaged on a Zeiss LSM900 confocal microscope using the Airyscan mode with a ×63/1.4 NA objective. The image underwent Airyscan processing with autofilter selected within Zeiss Zen. The processed image was then transferred to Imaris for volumetric reconstruction as described above.

### STED microscopy

Samples were stained for NG2 and VGluT2 and mounted using ProLong Diamond Antifade (Thermo Fisher Scientific, P36965) and coverslipped with No. 1.5H coverslips (Azer Scientific, ES0107222). Images were acquired on a Leica SPX8 STED microscope with a ×100/1.4 NA objective on Leica Application Suite X software. In Fig. 1j, single slices and maximum projections for two example OPCs are shown. For the maximum projections, some *z* sections were omitted due to imaging artifacts.

### pAAV:hSYN-synaptophysin-mCherry-eGFP (pSynDig)

We purchased the presynaptic ATP Syn-ATP reporter (Addgene, 51819) and the fluorescent marker hSYN–eGFP (Addgene, 50465) plasmids for downstream creation of the probe for synaptic digestion (pSynDig) construct. We amplified an elongated coding region for synaptophysin–mCherry from the Syn-ATP construct (Addgene, 51819; forward primer, 5′-GCGCAGTCGAGAAGGTACCGGCAGCAATGGACGTGGTG-3′, 5′-reverse primer: CCTTGCTCACCATGGTGGCGGGTCCCTTGTACAGCTCG-3-′). The elongated coding region (1,728 bp) was then gel-purified using the Qiagen MinElute Gel Extraction Kit (28604) and used in downstream Gibson assembly with the New England Biolabs HiFi DNA Assembly Cloning Kit (NEB, E5520S) to insert the amplified region into the hSyn–eGFP vector (Addgene, 50465) following its linearization with BamHI (NEB, R0136S). The resulting plasmid was gel-purified and sequenced before packaging into an adeno-associated virus (AAV) to yield AAV9-hSYN-pSynDig at a titer of 1.2 × 10^14^ genome copies per ml.

### pSynDig validation and quantification

We confirmed that pSynDig injections into the dLGN labeled VGluT2^+^ TC inputs in V1 by imaging mCherry^+^, eGFP^+^ and VGluT2^+^ puncta (following immunostaining with an antibody against VGluT2) in confocal images taken with a ×63 objective at Nyquist settings for downstream deconvolution using Huygens Essentials (SVI). Images were deconvolved based on imaging parameters (for example, pixel resolution, excitation wavelength, number of excitation photons, depth of acquisition, numerical aperture of objective and medium and emission wavelength), and the Huygens express deconvolution wizard was set to conservative deconvolution as a means of increasing image resolution and signal to noise. We measured the intensity of each channel using line intensity quantification in ImageJ. We observed largely overlapping mCherry and eGFP signal colocalized with VGluT2, as expected. We next measured the degree of colocalization between the eGFP and mCherry signals using the mean Pearson’s correlation coefficients across three images per animal. As expected, we observed a small percentage of puncta that were mCherry^+^ but eGFP^−^. To verify that these mCherry^+^ eGFP^−^ puncta represented inputs in the process of lysosomal degradation, we immunostained the tissue for the lysosomal marker Lamp2 and took 3D images (*z*-stacks) on a confocal microscope using a ×63 objective. In postprocessing, we applied a Gaussian blur of 0.132 µm. Maximum projections of the *z*-stacks were made, and then, the mean intensities of the mCherry, eGFP and Lamp2 channels were quantified. Images were then processed using Imaris, where lysosomes were reconstructed using a Surface object. In the wizard, the recorded mean intensities from ImageJ were used as the threshold value to define the surfaces. Masks of the eGFP and mCherry channels were made from the lysosome surfaces to analyze the within-lysosome pSynDig signal. Surfaces of the masked eGFP and mCherry signals were created, this time using 0.75 * (recorded mean intensity) as the threshold. To quantify the intensity of mCherry and eGFP outside of lysosomes, the masked channels were subtracted from the original channels, thereby removing any signal that was within a lysosome. mCherry and eGFP surfaces were generated from these subtracted channels, using the same wizard thresholds as for the within-lysosome group. The sum intensity statistic of the eGFP and mCherry signals was collected for the within- and outside-lysosome surfaces, before being normalized to the respective mCherry signal. The example image in Extended Data Fig. 8a was taken with a ×63 objective with ×1.5 digital zoom, before being deconvolved for clarity.

To quantify pSynDig in OPCs, the same approach was used as for the within-lysosome group, where OPC surfaces were reconstructed and used to mask the mCherry and eGFP channels. In Fig. 2f, only mCherry surfaces are shown and they are pseudocolored white or magenta if they overlapped with an eGFP surface or not, respectively.

### AAV injections

Mice aged between 2 and 6 months were injected with meloxicam (2.5 mg kg^−1^, subcutaneous) before being anesthetized using isoflurane (SomnoSuite, Kent Scientific; 3–5% induction, 1–2% maintenance). Once anesthetic depth was achieved, mice were placed onto a stereotaxic apparatus where body temperature was maintained using a heating pad. Mice were then unilaterally injected with either AAV9-hSYN-eGFP (Addgene viral prep 50465-AAV9) or AAV9-hSYN-pSynDig (500 µl with a flow rate of 50 nl min^−1^) into the right hemisphere dLGN (*x* = 2.15, *y* = −2.15 and *z* = −2.9 mm from bregma). Following surgery, animals were administered Flunixin (10 mg kg^−1^) and allowed to recover on a heating pad before returning to their home cages.

### Chronic window implantation

Mice (*n* = 10) aged between 2 and 6 months, previously injected with meloxicam (2.5 mg kg^−1^, subcutaneous) were anesthetized using isoflurane (3–5% induction, 1–2% maintenance), and body temperature was maintained with a heating pad throughout surgery and during initial recovery. After the initial AAV injection (as described above), a craniotomy of >3 mm in diameter was drilled using a dental drill (RWD, 78001) over V1 at approximately +2.5 mm lateral and −2.9 mm posterior from bregma. A 3-mm glass coverslip, sterilized with 70% ethanol, was then placed over the craniotomy and a mixture of surgical glue (Vetbond, 3M) and cyanoacrylate glue was used to secure the coverslip onto the skull. The skull was covered with a thin layer of Vetbond and then sealed with dental cement (Ortho-Jet, Land Dental). Finally, a custom-made head bar was secured onto the skull using luting cement (Metabond, C&B). Mice were then administered flunixin meglumine (10 mg kg^−1^, intraperitoneal), allowed to recover on a heating pad until ambulatory and then allowed to recover for 1 to 2 weeks before imaging. After recovery, the quality of the windows was checked before imaging, and mice with suboptimal windows were killed and used for downstream immunofluorescence quantification in fixed tissue.

### In vivo two-photon imaging

For in vivo imaging experiments, mice were secured into a custom head mount and movement restraint system before being placed onto the two-photon microscope. Mice were imaged using a custom two-photon system (Independent Neuroscience Services) with a ThorLabs tunable Tiberius laser. Laser wavelength was tuned to 980 nm to image both tdTomato and eGFP concurrently. We used a ×16/NA 0.8 water immersion lens (Nikon), and light was captured using two photomultiplier tubes fitted with filters (520–565 and >565 nm) for eGFP and tdTomato, respectively. While imaging at a depth between 150 and 350 μm from the pia, the laser power was kept below 30 mW to avoid photodamage. Imaging volumes were captured at near Nyquist settings (either 512 × 512 or 1,024 × 1,024 for time-lapse and single-time-point recordings, respectively; resulting in voxels ≤264 × 264 × 1,000 nm) and were selected for fields with tdTomato^+^ cells with distinguishable OPC morphology and TC inputs. For time-lapse recording, volumes were taken once per minute. Raw image files were then processed using Huygens software (SVI) for cross-talk correction, followed by registration along the *z* and *t* dimensions and processing with the multiphoton deconvolution wizard (set to conservative deconvolution as previously described for pSynDig experiments). Cross-talk-corrected, registered and deconvolved images were then imported into Imaris and Surface objects of OPCs and TC inputs were created, with surfaces of OPCs being limited to the OPC soma and major processes. TC inputs were then classified by their distance to the OPC’s surface using the ‘shortest distance to surfaces’ filter function in Imaris, with TC inputs that were greater than 0 nm from the OPC surface classified as noncontacting inputs, inputs with a distance to OPCs of 0 nm classified as contacting inputs and inputs with a distance of less than −270 nm classified as ‘engulfed’ inputs. The classified thalamocortical inputs’ volumes were then averaged per video and per time frame and normalized to the average volume of noncontacting inputs over the entire imaging period to avoid bleaching effects where applicable. For data presentation, the OPC surface volume was pseudocolored with green and classified input volumes were pseudocolored using the ‘overlapped volume ratio to surface’ function.

#### Note on identifying OPCs in vivo

Because we typically imaged NG2-CreER^T2^tdTomato mice more than 2 weeks after administration of TAM, some of the Cre-expressing OPCs had differentiated to mature oligodendrocytes labeled with tdTomato by the time of imaging. However, OPCs could be easily distinguished from oligodendrocytes based on their oblong, bean-shaped somata compared to oligodendrocytes, the somata of which were more spherical (Extended Data Fig. 1).

### Cell isolation and flow cytometry

Mice were first anesthetized with isofluorane and subsequently perfused transcardially with ice-cold PBS. Brains were then removed from the skull and the cortices were dissected and chopped into ~3-mm pieces for overnight enzymatic digestion with 0.5× Accumax (Thermo Fisher Scientific, SCR006). The tissue was further homogenized in a buffer solution (150 mM HEPES, 1× HBSS, 1% BSA, 2 mM EDTA and 5% glucose) by gentle pipetting using a 1-ml cut pipet-tip, and once again with a 1-ml uncut pipet-tip. OPCs and microglia were then enriched using a 40% Percoll in HBSS solution and centrifuged at 600*g* for 25 min. The cell pellet was washed and incubated with a CD16–CD32 receptor blocking antibody at 1:100 (Thermo Fisher Scientific, 14-0161-82) for 10 min. Next, the glial cells were stained with viability dye live/dead aqua stain at 1:1,000 (Thermo Fisher Scientific, L34957), and antibodies were directed against cell surface proteins for 30 min. The OPC population was identified using A2B5-AF488 at 1:100 (Thermo Fisher Scientific, FAB1416G) in combination with CD140a–PDGFRA-PE-Cy7 at 1:100 (BioLegend, 135912), and microglia were selected based on the expression of CD11b-PerCP-Cy5.5 at 1:100 (BioLegend, 101227) and CD45-Pacific Blue at 1:100 (BioLegend, 157211). To stain the intracellular synaptic material, the cells were first fixed with 1% PFA in HBSS for 10 min at room temperature, permeabilized with FoxP3/TRN (Life Technologies, 00-5523-00) staining buffer set according to the manufacturer’s protocol and stained with the following antibodies: SNAP25-AF 647 at 1:100 (BioLegend, 836311) or SYN1-AF 647 at 1:100 (Cell Signaling, 11127S). OPCs and microglia were analyzed with BD Dual Fortessa, and the results were analyzed using CytoexploreR^24^. For the gating strategy details, see Extended Data Fig. 6.

### Blinding

Experimenters were blinded to conditions for quantitative imaging experiments. One experimenter collected the tissue and assigned it a randomized label before providing the blinded tissue to another experimenter for analysis. After data acquisition and processing, the data were plotted in GraphPad by L.C., A.F. or Y.A. after which the samples were unblinded.

### Statistics and reproducibility

For all imaging datasets, we analyzed a minimum of three images over a set of three mice. For data computed based on the individual cells, multiple cells were taken from a minimum of three images per mouse (and a minimum of three mice per condition where applicable) and used as representative data points. Littermate controls were used whenever possible, for example, in the PLX experiments. For in vivo imaging, a minimum of three mice were used for analysis to create representative OPC volumes. Experimental mice were subject to exclusion based on window integrity as well as the signal-to-noise ratio (SNR) of fluorescent protein emissions. Both single volumes and time-lapse volumes were subject to exclusion based on the motion artifact as well as overt changes in SNR over the imaging session. Three independent replicates, with one mouse per condition within a given replicate, were used for flow cytometry datasets. Each independent replicate was analyzed separately and then pooled for data presentation.

Sample sizes and the number of mice/cells used were based on means and standard deviations found within our own lab and by others in similar experiments^2^. No statistical methods were used to predetermine the sample size.

All data are presented as the *n* representing individual cells or mouse averages, with exact *n* information in the figure legends. Parametric tests were used for Gaussian datasets, with nonparametric tests used when parametric tests were not applicable. Before unblinding and finalizing analyses, we removed outliers using the ‘Identify Outliers’ function in GraphPad (using the robust regression and outlier removal (ROUT) method with Q (maximum desired false discovery rate) = 1%). All statistical analyses were performed in GraphPad by L.C., A.F. or Y.A. with details described in the figure legends.

### Figure production

Representative images were rendered in either Imaris or ImageJ, where contrast and brightness were altered across the entire image for ease of viewing.

### Reporting summary

Further information on research design is available in the Nature Research Reporting Summary linked to this article.

## Online content

Any methods, additional references, Nature Research reporting summaries, source data, extended data, supplementary information, acknowledgements, peer review information; details of author contributions and competing interests; and statements of data and code availability are available at 10.1038/s41593-022-01170-x.

## Supplementary information

Reporting Summary

Supplementary Video 1 Example time-lapse recording of OPC–TC interactions.

Supplementary Video 2 Example OPC–TC input interaction with 3D reconstruction.

## Data Availability

Fixed raw images used in analysis, as well as for micrographs, and associated inhouse ImageJ macros used in Figs. 1–3 can be accessed in the following repository: 10.5281/zenodo.6991299. The raw and processed single-cell RNA-seq files are available at Gene Expression Omnibus under accession number GSE102827. All other data that support the findings of this study are available from the corresponding author on reasonable request.

## Code availability

ImageJ macros are included in the Zenodo data repository at 10.5281/zenodo.6991299.
