# Publisher Correction: Oligodendrocyte precursor cells engulf synapses during circuit remodeling in mice

**DOI:** 10.1038/s41593-022-01209-z

**Published:** 2022-11-07

**Authors:** Yohan S. S. Auguste, Austin Ferro, Jessica A. Kahng, Andre M. Xavier, Jessica R. Dixon, Uma Vrudhula, Anne-Sarah Nichitiu, Daniele Rosado, Tse-Luen Wee, Ullas V. Pedmale, Lucas Cheadle

**Affiliations:** 1 Cold Spring Harbor Laboratory, Cold Spring Harbor, NY USA; 2 School of Biological Sciences, Cold Spring Harbor Laboratory, Cold Spring Harbor, NY USA

**Keywords:** Glial biology, Synaptic development, Glial stem cells

Correction to: *Nature Neuroscience* 10.1038/s41593-022-01170-x, published online 28 September 2022.

In the version of this article initially published, the text in the seventh paragraph now reading “Furthermore, we used a viral probe for synaptic digestion, AAV9-hSYN-pSynDig^15^,” originally appeared as “…AAV9-hSYN-ExPre^15^.” The text has been amended in the HTML and PDF versions of the article.

