# Supplementary material for: Oligodendrocyte precursor cells engulf synapses during circuit remodeling in mice
Source: Nat Neurosci. 2022 Sep 28;25(10):1273–8. doi: 10.1038/s41593-022-01170-x (PMC9534756; doi:10.1038/s41593-022-01170-x)
Supplement: Supplementary file 1 — Reporting Summary [file 41593_2022_1170_MOESM1_ESM.pdf]

nature portfolio

Corresponding author(s): Lucas M. Cheadle, PhD

Last updated by author(s): Aug 16, 2022

Reporting Summary

Nature Portfolio wishes to improve the reproducibility of the work that we publish. This form provides structure for consistency and transparency in reporting. For further information on Nature Portfolio policies, see our Editorial Policies and the Editorial Policy Checklist.

Statistics

For all statistical analyses, confirm that the following items are present in the figure legend, table legend, main text, or Methods section.

n/a	Confirmed	
☐	☒	The exact sample size (*n*) for each experimental group/condition, given as a discrete number and unit of measurement
☐	☒	A statement on whether measurements were taken from distinct samples or whether the same sample was measured repeatedly
☐	☒	The statistical test(s) used AND whether they are one- or two-sided<br>*Only common tests should be described solely by name; describe more complex techniques in the Methods section.*
☐	☒	A description of all covariates tested
☐	☒	A description of any assumptions or corrections, such as tests of normality and adjustment for multiple comparisons
☐	☒	A full description of the statistical parameters including central tendency (e.g. means) or other basic estimates (e.g. regression coefficient) AND variation (e.g. standard deviation) or associated estimates of uncertainty (e.g. confidence intervals)
☐	☒	For null hypothesis testing, the test statistic (e.g. *F*, *t*, *r*) with confidence intervals, effect sizes, degrees of freedom and *P* value noted<br>*Give P values as exact values whenever suitable.*
☒	☐	For Bayesian analysis, information on the choice of priors and Markov chain Monte Carlo settings
☒	☐	For hierarchical and complex designs, identification of the appropriate level for tests and full reporting of outcomes
☐	☒	Estimates of effect sizes (e.g. Cohen's *d*, Pearson's *r*), indicating how they were calculated

Our web collection on statistics for biologists contains articles on many of the points above.

Software and code

Policy information about availability of computer code

Data collection	Software: Zen Black 2012 and 2011, DeltaVision(OMx3.70.9220.0), LasX(3.5.7.23225), and ScanImage(ScanImage Basic 2020.1.4) were used to acquire images. BD FACSDiva 6 was used for flow cytometry.
Data analysis	ImageJ2 version 2.3.0/1.53f, Imaris version 9.8.0, Huygens Essentials version 21.10, Graphpad Prism version 9.2.0, CytoExplore R package version 1.1.0

For manuscripts utilizing custom algorithms or software that are central to the research but not yet described in published literature, software must be made available to editors and reviewers. We strongly encourage code deposition in a community repository (e.g. GitHub). See the Nature Portfolio guidelines for submitting code & software for further information.

Data

Policy information about availability of data

All manuscripts must include a data availability statement. This statement should provide the following information, where applicable:

- Accession codes, unique identifiers, or web links for publicly available datasets
- A description of any restrictions on data availability
- For clinical datasets or third party data, please ensure that the statement adheres to our policy

The majority of source data are uploaded to the Zenodo repository (DOI# 10.5281/zenodo.6991299) and will become publicly available upon publication. See full data availability statement in revised manuscript.

Field-specific reporting

Please select the one below that is the best fit for your research. If you are not sure, read the appropriate sections before making your selection.

☒ Life sciences ☐ Behavioural & social sciences ☐ Ecological, evolutionary & environmental sciences

For a reference copy of the document with all sections, see nature.com/documents/nr-reporting-summary-flat.pdf

Life sciences study design

All studies must disclose on these points even when the disclosure is negative.

Sample size

Sample sizes were based off of Schafer et al. (2012) and prior experience with these approaches.

Data exclusions

One data point in the P20 microglial engulfment of VGluT2 (Fig. 1D) was a strong outlier (greater than 3 times the interquartile range) and therefore excluded. We also excluded strong outliers from the data shown in Figure 1F,L, and Extended Data Figure 3B by using the Graphpad Prism function "Identify outliers" based upon the ROUT method (robust regression and outlier removal) with a Q (maximum desired false discovery rate) = 1 %. Outlier detection and removal was decided a priori as a means of providing more accurate data given the potential for noise in quantifying fluorescence imaging data.

Replication

For all imaging datasets, we have analyzed a minimum of three images per mouse over a set of three mice. For data where individual cells are plotted, multiple cells were taken from a minimum of three images per mouse (and a minimum of three mice per condition where applicable) and used as representative data points. Furthermore, littermate controls were used whenever possible. For in vivo imaging, a minimum of three mice were used for analysis to create representative oligodendrocyte precursor cell (OPC) volumes. In vivo imaging mice were subject to exclusion based off of window integrity as well as signal to noise (SNR) of fluorescent protein emissions. Both single volumes and time-lapse volumes were subject to exclusion based off of motion artifact as well as overt changes to SNR over the imaging session. For flow cytometry datasets three independent replicates with one mouse per condition within a given replicate were used. Each independent replicate was analyzed separately and then pooled for data presentation.

Randomization

Mice were either selected for the study because of correct genotype or simply ordered from Jackson Laboratory. Mice were randomly assigned to groups while ensuring an even distribution of sexes.

Blinding

Experimenters were blinded to conditions for quantitative imaging experiments. One experimenter harvested the tissue and assigned it a randomized label before providing the blinded tissue to another experimenter for analysis. After data acquisition and processing, the data were plotted in Graphpad and then samples were unblinded.

Reporting for specific materials, systems and methods

We require information from authors about some types of materials, experimental systems and methods used in many studies. Here, indicate whether each material, system or method listed is relevant to your study. If you are not sure if a list item applies to your research, read the appropriate section before selecting a response.

Materials & experimental systems

n/a	Involved in the study	
☐	☒	Antibodies
☒	☐	Eukaryotic cell lines
☒	☐	Palaeontology and archaeology
☐	☒	Animals and other organisms
☒	☐	Human research participants
☒	☐	Clinical data
☒	☐	Dual use research of concern

Methods

n/a	Involved in the study	
☒	☐	ChIP-seq
☐	☒	Flow cytometry
☒	☐	MRI-based neuroimaging

Antibodies

Antibodies used

Primary antibodies: rat α NG2 (Thermo Fisher Scientific: MA5-24247) lot WJ3410656; guinea pig α Vglut2 (Sigma: AB2251-I) lot 3482777; rabbit α Iba1 (Wako: 019-19741); rat α Lamp2 (Abcam: AB13524) lot GR3245901-20; rabbit α Sox10 (Abcam: AB227680) lot GR3252221-7, chicken α Homer1b/c (Synaptic Systems: 160026); chicken α GFAP (Abcam: ab4674); rat α MBP (Abcam: ab7349); rabbit α LRP1 (Abcam ab92544) lot GR259330-49; rabbit α EEA1 (Abcam ab2900) lot GR3365695-4; rabbit α Rab7 (Abcam: ab137029) lot GR155792-42; CD16/CD32 receptor blocking antibody (Thermo Fisher Scientific: 14–0161-82) lot 2293306; A2B5-AF488 (R&D systems: FAB1416G) lot AFHP0221071; CD140a/PDGFRA-PE-Cy7 (Biolegend: 135912) lot B329879; CD11b-PerCP-Cy5.5 (Biolegend: 101227) lot B314770; VGLUT2-AF 647 (Novus Biologicals: NBP2-59330AF647) lot D105711; SNAP25-AF 647 (Biolegend: 836311) lot B298057 ; SYN1-AF 647(Cell Signaling: 11127S) lot 3.

Secondary antibodies from Invitrogen: goat α guinea pig 647 (A21450); goat α guinea pig 555 (A21435) lot 2180698; donkey α rabbit 405 (A21450) lot 2273716; goat α rabbit 488 (A11008) lot 2147635; goat α rabbit 647 (A21428) lot 2359136; goat α rat 405 (A48261)

lot VJ314973; donkey α rat 488 (A21208) lot 2273677; goat α rat 647 (A21247) lot 2219256; goat α guinea pig 555 (A21435); goat α guinea pig 488 (A11073)

Validation

Commercial antibodies were validated by the manufacturers and or used in references found within links below:
rat α NG2: https://www.thermofisher.com/antibody/product/MA5-24247.html?gclid=CjwKCAjw3K2XBhAzEiwAmmgrAr3PQHdapqYFJyNZKvUUt6G51nUoXNzTPimto0fdNJAmND3SGYTCkRoCAyAQAvD_BwE&ef_id=CjwKCAjw3K2XBhAzEiwAmmgrAr3PQHdapqYFJyNZKvUUt6G51nUoXNzTPimto0fdNJAmND3SGYTCkRoCAyAQAvD_BwE:G:s&s_kwcid=AL!3652!3!459736943987!!!g!!&cid=bid_pca_aup_r01_co_cp1359_pjt0000_bid00000_0se_gaw_dy_pur_con

guinea pig α Vglut2: https://www.sigmaaldrich.com/US/en/product/mm/ab2251i?gclid=CjwKCAjw3K2XBhAzEiwAmmgrAp3ZKF0YcZbWO8WPC4lvj8cDkim4U4DtD2aJ2l9w1lbTm2yYCV9VxxoC3IQQAvD_BwE

rabbit α Iba1: https://labchem-wako.fujifilm.com/us/product/detail/W01W0101-1974.html
rat α Lamp2: https://www.abcam.com/lamp2-antibody-gl2a7-ab13524.html?productWallTab=ShowAll

rabbit α Sox10: https://www.abcam.com/sox10-antibody-sp267-ab227680.html?productWallTab=ShowAll
chicken α Homer1b/c: https://sysy.com/product/160026

chicken α GFAP: https://www.abcam.com/gfap-antibody-ab4674.html?productWallTab=ShowAll
rat α MBP: https://www.abcam.com/myelin-basic-protein-antibody-12-ab7349.html?productWallTab=ShowAll

rabbit α LRP1: https://www.abcam.com/lrp1-antibody-epr3724-ab92544.html
rabbit α EEA1: https://www.abcam.com/eea1-antibody-early-endosome-marker-ab2900.html

rabbit α Rab7: https://www.abcam.com/rab7-antibody-epr7589-ab137029.html

CD16/CD32 receptor blocking antibody https://www.thermofisher.com/antibody/product/CD16-CD32-Antibody-clone-93-Monoclonal/14-0161-82

A2B5-AF488 https://www.rndsystems.com/products/human-mouse-rat-chicken-a2b5-alexa-fluor-488-conjugated-antibody-105_fab1416g

CD140a/PDGFRA-PE-Cy7 https://www.biolegend.com/de-de/products/pe-cyanine7-anti-mouse-cd140a-antibody-14822

CD11b-PerCP-Cy5.5 https://www.biolegend.com/de-de/products/percp-cyanine5-5-anti-mouse-human-cd11b-antibody-4257

CD45-Pacific Blue https://www.biolegend.com/en-us/products/pacific-blue-anti-mouse-cd45-antibody-19250 VGLUT2-AF 647 https://www.novusbio.com/products/vglut2-antibody-s29-29_nbp2-59330af647

SNAP25-AF https://www.biolegend.com/en-gb/search-results/alexa-fluor-647-anti-snap-25-antibody-18600

SYN1-AF 647 https://www.cellsignal.com/products/antibody-conjugates/synapsin-1-d12g5-xp-rabbit-mab-alexa-fluor-647-conjugate/11127

Animals and other organisms

Policy information about studies involving animals; ARRIVE guidelines recommended for reporting animal research

Laboratory animals

Mus musculus, C57Bl/6J, B6.Cg-Tg(Cspg4-cre/Esr1*), BAkik/J, B6.Cg-Gt(ROSA)26Sortm14(CAG-tdTomato)Hze/J. Fixed tissue analyses were performed on equal numbers of male and female mice at postnatal days (P)10, P20, P27, and P90. Live imaging was performed on animals between 2 and 6 months of age.

Wild animals

The study did not involve wild animals.

Field-collected samples

The study did not involve animals collected from the field.

Ethics oversight

Institutional Animal Care and Use Committee at Cold Spring Harbor Laboratory, protocol # 20-3

Note that full information on the approval of the study protocol must also be provided in the manuscript.

Flow Cytometry

Plots

Confirm that:

- [x] The axis labels state the marker and fluorochrome used (e.g. CD4-FITC).
- [x] The axis scales are clearly visible. Include numbers along axes only for bottom left plot of group (a 'group' is an analysis of identical markers).
- [x] All plots are contour plots with outliers or pseudocolor plots.
- [x] A numerical value for number of cells or percentage (with statistics) is provided.

Methodology

Sample preparation	Mice were first anesthetized with isofluorane and subsequently perfused transcardially with ice cold PBS. Brains were then removed from the skull and the cortices dissected and chopped into ~3 mm pieces for overnight enzymatic digestion with 0.5X Accumax (Thermo Fisher Scientific: SCR006). The tissue was further homogenized in a buffer solution (150 mM HEPES, 1X HBSS, 1% BSA, 2 mM EDTA and 5% Glucose) by gentle pipetting using a 1 mL cut pipet-tip, and once again with a 1 mL uncut pipet-tip. OPCs and microglia were then enriched using a 40% Percoll in HBSS solution and centrifuged at 600 g for 25 mins. The cell pellet washed and incubated with a CD16/CD32 receptor blocking antibody at 1:100 (Thermo Fisher Scientific: 14–0161-82) for 10 min. Next, the glial cells were stained with viability dye Live/Dead aqua dead stain at 1:1000 (Thermo Fisher Scientific: L34957) and antibodies directed against cell surface proteins for 30 min. The OPC population was identified using A2B5-AF488 at 1:100 (Thermo Fisher Scientific: FAB1416G) in combination with CD140a/PDGFRA-PE-Cy7 at 1:100 (Biolegend: 135912) and microglia were selected based on the expression of CD11b-PerCP-Cy5.5 at 1:100 (Biolegend: 101227) and CD45-Pacific Blue at 1:100 (Biolegend 157211). In order to stain the intracellular synaptic material, the cells were first fixed with 1% paraformaldehyde in HBSS for 10 min at RT, permeabilized with FoxP3/TRN (Life Technologies: 00-5523-00) staining buffer set according to the manufacturer's protocol and stained with the following antibodies: VGLUT2-AF 647 at 1:100 (Novus Biologicals: NBP2-59330AF647) , SNAP25-AF 647 at 1:100 (Biolegend: 836311), or SYN1-AF 647 antibodies at 1:100 (Cell Signaling 11127S)
Instrument	BD LSRFortessa
Software	The BD FACSDiva was used to collect the data and CytoexplorerR was used to analyze and generate the the plots and UMAP.
Cell population abundance	Nothing was sorted and/or collected
Gating strategy	The cells were initially distinguished from debris by the expression of CD45 and PDGFRA proteins. Next, the CD45lo and PDGFRAhi population were separately gated as follows: single cells1 → single cells2 → viable cells → OPCs (PDGFRAhi A2B5hi) and microglia (Cd11bhi CD45lo). An independent fluorescence minus one (FMO) control was used as a baseline to set the gate for the negative and positive events related to SYNAPSIN and SNAP25. The highest mean fluorescence intensity in the microglia population was used to set the gate for the high positive events in OPCs.

☒ Tick this box to confirm that a figure exemplifying the gating strategy is provided in the Supplementary Information.
